# A review of recent *Cryptosporidium hominis* and *Cryptosporidium parvum gp60* subtypes

**DOI:** 10.1016/j.crpvbd.2025.100292

**Published:** 2025-07-06

**Authors:** Deborah B. Oladele, Martin Swain, Guy Robinson, Amanda Clare, Rachel M. Chalmers

**Affiliations:** a Department of Life Sciences, Aberystwyth University, Penglais, Aberystwyth, Ceredigion, SY23 3DA, UK; b Cryptosporidium Reference Unit, Public Health Wales Microbiology, Singleton Hospital, Sgeti, Swansea, SA2 8QA, UK; c Swansea University Medical School, Singleton Park, Swansea, SA2 8PP, UK

**Keywords:** *Cryptosporidium*, *gp60*, Subtyping, NHP, Zoonotic, Anthroponotic, Apicomplexa

## Abstract

*Cryptosporidium* spp. are known to cause gastroenteritis (cryptosporidiosis) in numerous hosts, including humans. Understanding the diversity within this genus of parasites requires accurate subtyping, which is frequently performed by sequencing part of the *gp60* (60-kDa glycoprotein) gene. This literature review examines *Cryptosporidium hominis* and *Cryptosporidium parvum gp60* subtypes reported between December 2018 and January 2024 in humans, livestock, and non-human primates (NHPs). The review highlights emerging trends in the subtypes reported and reveals the shifting dominance of subtype families, which can be influenced by factors such as anthroponotic interactions. The *C. parvum* IIa and IId families remain major contributors to infections across a variety of hosts, with recent reports indicating the continued emergence of the IId family. Furthermore, previously established and newly reported subtypes detected in NHPs highlight the potential for genetic recombination between human-adapted and NHP-adapted subtypes.

## Highlights

• A review of recently reported *C. hominis* and *C. parvum gp60* subtypes is provided.
• 264 *gp60* subtypes were reported from 1st December 2018 to 31st January 2024.
• Emerging subtypes and shifting dominance of subtype families are identified.
• Newly identified subtypes in humans, livestock and NHPs indicate potential zoonotic transmission risks.

## Graphical abstract

**Figure:**
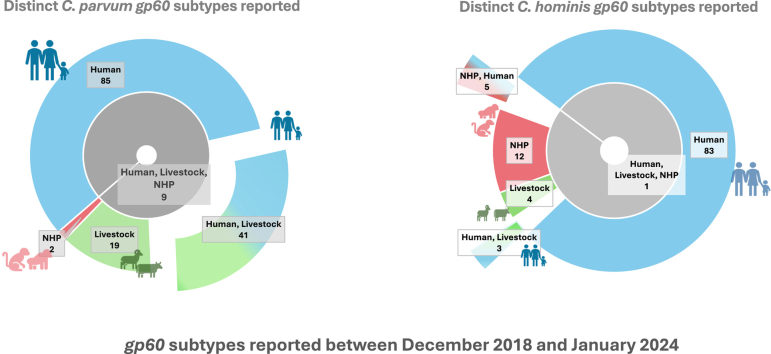

## 1 Introduction

Apicomplexan enteric protozoan parasites in the genus *Cryptosporidium* are known to cause gastroenteritis (cryptosporidiosis) in a wide range of hosts, including humans (Gerace et al., 2019). *Cryptosporidium* spp. infections are of particular concern in low- and middle-income countries, where there is a substantial burden in young children (Hajissa et al., 2022). In high-income countries, *Cryptosporidium* spp. pose a public health threat through the occurrence of outbreaks that can be widespread. The parasites are transmitted *via* a faecal-oral route by the ingestion of oocysts either directly from infected people or animals, or through contaminated food, water or surfaces (Gerace et al., 2019). A continually growing list of at least 44 species and over 120 genotypes of *Cryptosporidium* have been identified, each exhibiting a spectrum of host specificity that varies both in terms of range and preference (Ryan et al., 2021a).

Two species, *Cryptosporidium hominis* and *Cryptosporidium parvum*, account for the majority of human cryptosporidiosis cases. *Cryptosporidium parvum* is zoonotic, infecting a wide range of hosts, including major domestic livestock species, whereas *C. hominis* is predominantly anthroponotic (Cacciò and Chalmers, 2016). *Cryptosporidium* oocysts are the diagnostic target, but the challenge posed by the lack of significant morphological differences between species has necessitated the use of genotyping for accurate species identification. Genetic markers with sufficient polymorphism, such as the small-subunit ribosomal RNA (*SSU* rRNA), *Cryptosporidium* outer wall protein (*cowp*) and 70-kDa heat shock protein (*hsp70*) genes, became instrumental in discriminating *Cryptosporidium* species through standard PCR techniques, often utilising restriction fragment length polymorphisms (RFLP) or Sanger sequencing (Xiao and Ryan, 2004). The *SSU* rRNA gene was specifically targeted for its presence in multiple copies (5 per sporozoite and 20 per oocyst), facilitating the development of sensitive assays (Le Blancq et al., 1997; Jiang et al., 2005). More recently, real-time PCRs specifically identifying *C. parvum* and *C. hominis* have been published (Robinson et al., 2020). To further discriminate within *Cryptosporidium* species, sequencing part of the *gp60* (60-kDa glycoprotein) gene has been widely adopted as it is highly polymorphic and has provided a reasonable resolution for understanding the genetic diversity and evolutionary relationships of *Cryptosporidium* (Widmer and Lee, 2010; Xiao and Feng, 2017). This gene, responsible for encoding surface glycoproteins that play a role in host cell attachment and invasion, is sequenced and analysed in the region downstream of the N-signal peptide sequence which contains poly-serine (TCA/TCG/TCT) tandem repeats in most species and other highly variable regions (Strong et al., 2000; O’Leary et al., 2021).

In general, *gp60* subtype nomenclature involves the use of Roman numerals, lower-case and upper-case letters, and Arabic numbers. A full description, including the more unusual features and variations, is available in Robinson et al. (2025). The determination of *gp60* subtypes has been facilitated recently by CryptoGenotyper, a bioinformatic tool described by Yanta et al. (2021).

The *gp60* marker emerged as a pivotal genetic tool in the study of *Cryptosporidium* spp., particularly *C. parvum* and *C. hominis.* It aided epidemiological investigation and outbreak characterization and was also essential for distinguishing between zoonotic and anthroponotic families, especially within the diverse families of *C. parvum* (Chalmers et al., 2019; Nader et al., 2019).

However, the hypervariability within the *gp60* gene and its early evolution compared to the rest of the genome, have both aided and hindered its use as a genotyping tool (Abal-Fabeiro et al., 2013). For instance, in species other than *C. hominis* and *C. parvum,* which have received less epidemiological focus, amplification and subtyping of the *gp60* region are often hindered by variations in commonly used primer sites or the absence of the poly-serine tract in some species (Rojas-Lopez et al., 2020). These variations and substitutions in nucleotide sequences have resulted in a plethora of families and subtypes, particularly in *C. parvum*, and the nomenclature has evolved to capture newly discovered features, but occasionally resulting in confusion (Robinson et al., 2025).

Thus, it remains crucial to establish a standardized *gp60* nomenclature and update current knowledge of *gp60* subtypes. The recent manual by Robinson et al. (2025) provides guidelines for *gp60* subtype nomenclature. Building on this, our study aims to update current knowledge of reported *C. hominis* and *C. parvum gp60* subtypes to elucidate current subtype trends. This is important for maintaining the reliability of *gp60* in One Health surveillance, spillover-potential tracking and informing outbreak responses. We conducted a literature review to update and incorporate recent reports and trends of *C. hominis* and *C. parvum gp60* subtypes in humans, livestock and non-human primates (NHPs), described between December 2018 and January 2024.

## 2 Literature review search strategy

The review was limited to studies published between 1st December 2018 and 31st January 2024 to update previous reviews and ensure the inclusion of more recent data (Ryan et al., 2021a, 2021b; Chen et al., 2023). This timespan covered the period where an effect of the COVID-19 pandemic and interventions has been reported on *Cryptosporidium* cases (Knox et al., 2021; Adamson et al., 2023) and on the *Cryptosporidium gp60* subtypes in human cryptosporidiosis (Bacchetti et al., 2023).

### 2.1 Search strategy

The literature search was performed using PubMed, employing the search terms “*Cryptosporidium*” and “*gp60*” or “60 kDa glycoprotein”. The search targeted studies published between the 1st December 2018 and 31st January 2024, focusing on *C. parvum* and *C. hominis gp60* subtypes reported in humans, livestock, and NHPs. The initial search returned 222 results.

### 2.2 Screening criteria

A three-level screening process was applied. At the first level, a total of 73 publications were excluded based on the following criteria:

• Non-host studies: Studies involving animals other than humans, livestock, or NHPs) were excluded. Livestock were defined according to the definition of Encyclopaedia Britannica (2019), with additional inclusion of alpacas, rabbits, and poultry. The livestock considered were cattle, sheep, pigs, goats, horses, donkeys, mules, deer, buffalo, oxen, llamas, camels, poultry, alpacas, and rabbits. Studies involving reptiles, fishes, rodents, squirrels, pets (cats, dogs, hamsters, guinea pigs, ponies), pigeons, crows, minks, and ostriches were excluded.
• Publications focused solely on the development of newly reported protocols, diagnostics, or molecular assays without primary or secondary occurrence or epidemiological data were excluded.
• Studies concentrating exclusively on experimental parasitology or molecular biology without *Cryptosporidium gp60* subtype identification were also excluded.
• Studies that solely examined environmental samples were excluded.

At the second level, an additional 21 publications were excluded for not reporting or identifying infections caused by *C. parvum* or *C. hominis.* At the third level, 5 meta-analysis/systematic reviews were removed. This resulted in a preliminary total of 123 eligible publications. Additionally, 4 studies were excluded**.** Three studies passed the primary criteria but did not report any *gp60* subtypes. This was due either to the absence of *C. parvum* or *C. hominis* in the sampled hosts or to unsuccessful genotyping. One study was inaccessible due to closed access and being published in a non-English language. Finally, 119 publications were included for data extraction (see Supplementary file 1).

### 2.3 Details extracted

For every characterized *gp60* subtype reported in a study, the following were recorded:

• *Cryptosporidium* species, *gp60* family and *gp60* subtype. Samples with no subtype expressed in the paper but had available sequences were included and recorded.
• GenBank accession number if available or accession number from other databases.
• Whether newly reported (i.e. no previous report of the subtype) or not.
• For newly reported subtypes, extra information about the serine repeats and any additional repeats. Available sequences for the newly reported subtypes were verified on NCBI GenBank to confirm the subtype followed the naming convention promoted by Robinson et al. (2025). The sequences were imported to NCBI BLAST to confirm that there was no previous report prior to this study.
• Whether reported in an outbreak or not, as defined by the source publication.
• For a subtype reported in an outbreak, details of the transmission route, vehicle and setting were documented, when available. If the subtype was involved in multiple outbreaks within the same report, the setting for each was noted in the entry record.
• Infected host species; when possible, we employed the use of singular and plural forms to characterize whether a subtype was identified in one or more cases.
• Genotyping technique used for molecular characterization of the subtype.
• Country and WHO region of the origin of the sample inferred from the location of the study.
• Year of the study or investigation and year of publication.

### 2.4 Data handling

The *gp60* families and subtypes were enumerated and categorized by study, host, country, and WHO region.

## 3 Recently reported *gp60* subtypes in *Cryptosporidium hominis* and *C. parvum*

**Fig. 1:**
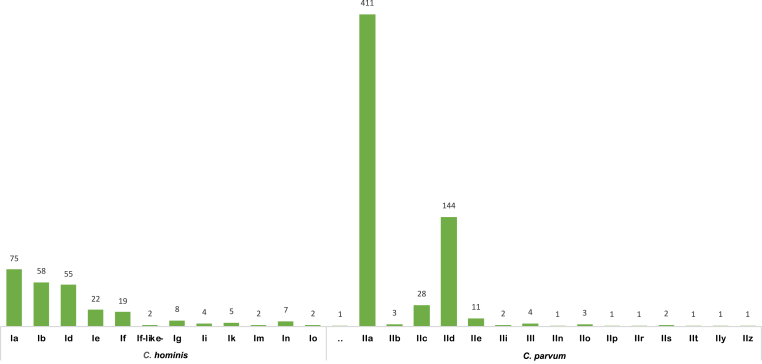
*Cryptosporidium hominis* and *C. parvum gp60* subtypes reported between December 2018 and January 2024 grouped by family. *Cryptosporidium parvum* families IIa and IId show the highest number of subtype reports during this period. The undesignated *C. parvum* family (denoted by “.“) for which only a part of the sequence is available, was found in Egypt, and the subtype family is still under confirmation.

The review identified a total of 879 *gp60* reports, with 264 distinct *gp60* subtypes (Supplementary file 2). A total of 12 *C. hominis gp60* families and 16 *C. parvum gp60* families were reported (Fig. 1; Table 1). Of the 264 distinct subtypes identified, 108 belonged to *C. hominis* and 156 to *C. parvum. Cryptosporidium hominis* families Ie, Id and If along with *C. parvum* families IIa and IId were reported in all host categories (Table 1).

**Table 1:** Distinct *C. hominis* and *C. parvum gp60* subtypes identified in reports between December 2018 and January 2024. More subtypes were identified in human hosts influenced by more epidemiological focus and higher report cases in humans along with challenges that can arise during infection source tracing.

Species	Family	Host category	Distinct *gp60* subtypes
*C. hominis*	Ia	Human	40
		Human, NHP	2
		NHP	1
		*Total*	*43*
	Ib	Human	10
		Human, NHP	1
		NHP	1
		*Total*	*12*
	Id	Human	17
		Human, Livestock	2
		Human, NHP	1
		*Total*	*20*
	Ie	Human	2
		Human, NHP, Livestock	1
		*Total*	*3*
	If	Human	7
		Livestock, Human	1
		NHP	1
		*Total*	*9*
	If-like-	Livestock	1
	Ig	Human	5
	Ii	NHP, Human	1
	Ik	Human	1
		Livestock	3
		*Total*	*4*
	Im	Human	1
		NHP	1
		*Total*	*2*
	In	NHP	6
	Io	NHP	2
Total *C. hominis*			108

*C. parvum*	IIa	Human	30
		Human, Livestock	31
		Human, NHP, Livestock	7
		Livestock	14
		*Total*	*82*
	IIb	Human	3
	IIc	Human	9
		Human, Livestock	2
		*Total*	*11*
	IId	Human	24
		Human, Livestock	8
		Livestock	4
		Livestock, Human, NHP	2
		*Total*	*38*
	IIe	Human	6
	IIi	Human	2
	IIl	Human	2
		Livestock	1
		*Total*	*3*
	IIn	Human	1
	IIo	Human	2
		NHP	1
		*Total*	*3*
	IIp	NHP	1
	IIr	Human	1
	IIs	Human	1
	IIt	Human	1
	IIy	Human	1
	IIz	Human	1
	.	Human	1
Total *C. parvum*			156

*Abbreviations*: NHP, non-human primate; *gp60*, 60 kDA glycoprotein.

**Fig. 2:**
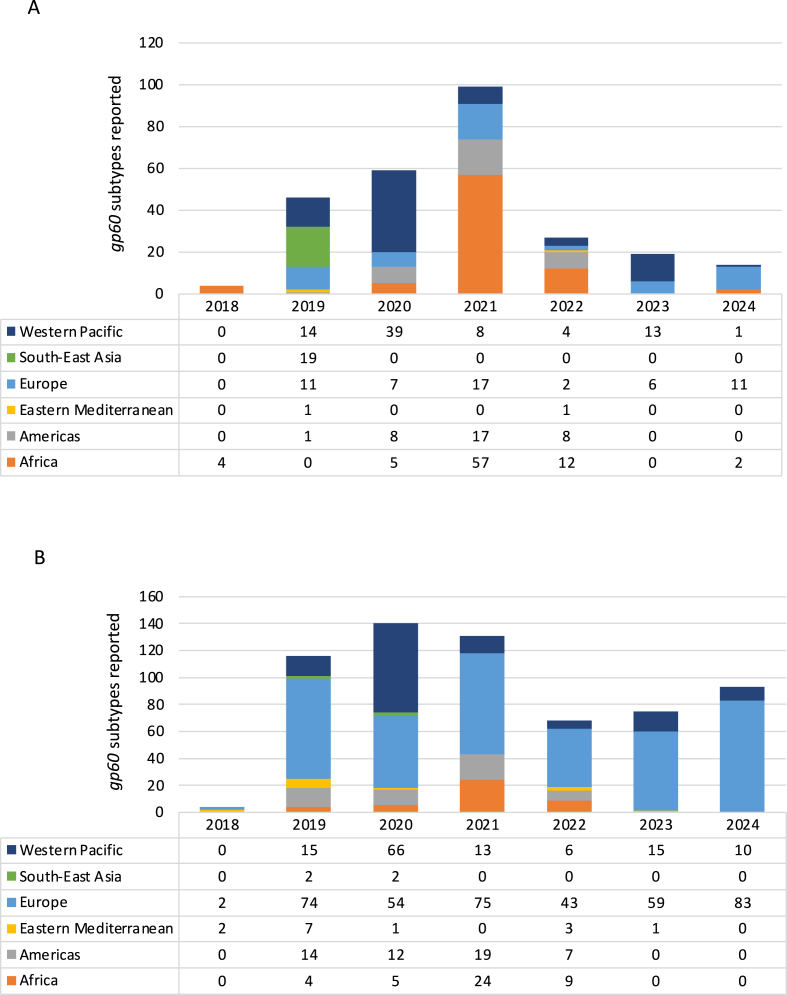
Reported *C. hominis* (**A**) and *C. parvum* (**B**) *gp60* subtypes by study publication year (December 2018 to January 2024) and WHO region where the study was carried out.

The number of subtypes reported varied by WHO region (location of study) and by year (Fig. 2). High numbers of *gp60* subtypes, especially *C. parvum* subtypes*,* were reported in Europe during this period. The number of reports observed from Europe in 2021, was considerably influenced by the publication of historical cases reported from Sweden/Scandinavia (Lebbad et al., 2021) (Fig. 2A and B). Examination of the data without these reports showed a notable decline in reports in 2021 and 2022, particularly for *C. hominis*, for which there were no reports in 2021 and only two in 2022 from France and Switzerland (Supplementary file 2).

In the Western Pacific, published reports for both *Cryptosporidium* spp. spiked in 2020 (Fig. 2A and B). Interestingly, the highest numbers of reports for both *Cryptosporidium* spp. in 2021 were recorded for Africa and the Americas. The Eastern Mediterranean and South-East Asia had the lowest numbers of reports, possibly due to underreporting and limited genotyping practices. (Fig. 2A and B; Supplementary file 2).

## 4 Prevalence, distribution, and host-association patterns of *Cryptosporidium hominis* and *C. parvum gp60* subtypes

A range of *gp60* subtype families were reported in human infections (Table 1), and their prevalence and distribution varied across geographical regions, likely influenced by factors such as differences in transmission dynamics, environmental conditions, socio-economic contexts, and diagnostic testing, subtyping and reporting practices (Xiao, 2010). The ten most frequently reported subtypes included two *C. hominis* and eight *C. parvum* subtypes (Supplementary file 2) and showed a wide variety of host infectivity with seven subtypes (IeA11G3T3, IIaA15G2R1, IIaA16G2R1, IIaA17G1R1, IIaA17G2R1, IIdA15G1, and IIdA19G1) reported in all three categories of hosts (humans, livestock, and NHPs). Nine of the ten subtypes were reported in both humans and livestock. Only reports of *C. hominis* IbA10G2 were restricted to humans and NHPs (Table 2, Table 3).

**Table 2:** *Cryptosporidium hominis gp60* subtypes reported in outbreaks. The IbA10G2 subtype was the most frequently reported, appearing in five different studies and multiple outbreaks.

*gp60* subtype	Outbreak setting/Vehicle/Source	Genotyping technique	GenBank ID	Host	Country	Study period	Reference
IaA14R3	Water-borne outbreaks (*n* = 3) from swimming pools in 2013, 2014 and 2015	Sanger	MK391438	Humans	England	2009–2017	Chalmers et al. (2019)
IaA17R3	Water-borne outbreak from a swimming pool (2019)	Sanger, NGS	MT952949	Human	Australia	2019–2020	Braima et al. (2021)
IaA20R3	Water-borne outbreak from a swimming pool in 2014	Sanger	MK391439	Humans	England	2009–2017	Chalmers et al. (2019)
IbA10G2	Water-borne outbreaks (*n* = 28) from swimming pools in 2011, 2012, 2013 (*n* = 4), 2014 (*n* = 6), 2015 (*n* = 7), 2016 (*n* = 7), and 2017, drinking water in 2013 (subtype also found in source water). Anthroponotic outbreak in a daycare nursery in 2015. Unknown source national outbreaks (*n* = 3) in 2012, 2015 and 2016	Sanger	MK391440	Humans	England and Wales	2009–2017	Chalmers et al. (2019)
IbA10G2	Water-borne outbreak from a swimming pool (2019)	Sanger, NGS	MT952950	Human	Australia	2019–2020	Braima et al. (2021)
IbA10G2	Water-borne outbreak from tap water consumption	Sanger	OK032157	Humans: children, immunocompromised adult, soldiers	French Guiana	2018	Menu et al. (2022)
IbA10G2	Water-borne outbreaks (*n* = 2) from water supply in military camps	Sanger		Humans	France	2017	Watier-Grillot et al. (2022)
IbA10G2	Water-borne outbreaks from swimming pools (2010, 2013, 2017), also anthroponotic outbreak in childcare centre (2017) and unknown causes (2010, 2013)	TIDE		Humans	New Zealand	2010–2021	Garcia-R and Hayman, 2023
IbA12G3	Water-borne outbreak from a swimming pool (2017)	Sanger	MK391441	Humans	England	2009–2017	Chalmers et al. (2019)
IbA12G3	Water-borne outbreaks (at least 3) from recreational aquatic centres (2020)	Sanger, NGS	MT952947	Humans	Australia	2019–2020	Braima et al. (2021)
IbA9G3	Water-borne outbreak in male child and adult after swimming (2019)	Sanger, NGS	MT952955	Humans: child, adult	Australia	2019–2020	Braima et al. (2021)
IdA14^a^	Water-borne outbreak in a swimming pool (2019) and unspecified outbreak in 2020. Two of the cases were acquired overseas from unknown sources	Sanger, NGS	MT952954	Humans	Australia	2019–2020	Braima et al. (2021)
IdA15G1^b^	Water-borne outbreak from a swimming pool (2019) and unspecified outbreak in 2020. Two of the cases were acquired overseas from unknown sources	Sanger, NGS	MT952951	Humans	Australia	2019–2020	Braima et al. (2021)
IdA16	Water-borne outbreak from a swimming pool (2016)	Sanger	MK391442	Humans	England	2009–2017	Chalmers et al. (2019)
IdA18	Water-borne outbreak from drinking water in 2013	Sanger	MK391443	Sheep: lambs	England	2009–2017	Chalmers et al. (2019)
IdA25	Water-borne outbreak from a swimming pool (2014)	Sanger	MK391444	Humans	England	2009–2017	Chalmers et al. (2019)
IeA12G3T3	Unspecified source outbreak in 13-year-old male (2020)	Sanger, NGS	MT952952	Human: child	Australia	2019–2020	Braima et al. (2021)
IgA17	Water-borne swimming pool outbreak (2013), unknown cause (2013)	TIDE		Humans	New Zealand	2010–2021	Garcia-R and Hayman (2023)
IgA20	Anthroponotic outbreak in childcare centre (2017)	TIDE		Humans	New Zealand	2010–2021	Garcia-R and Hayman (2023)
IiA17	Outbreak affecting father and son travelling together in 2013	Sanger		Humans	Sweden	2013–2014	Lebbad et al. (2021)
ImA13G1^c^	Water-borne outbreak involving swimming water between February and April 2022 amongst military personnel	Sanger	OP699729	Humans	Kenya	2022	Toriro et al. (2024)

*Abbreviations*: *gp60*, 60 kDA glycoprotein; NGS, Next Generation Sequencing; TIDE, Tracking of Indels by Decomposition.

a Mixed infection with IdA15G1.

b Mixed infection with IdA14. Subtype detected by NGS and not Sanger.

c Newly reported.

**Table 3:** *Cryptosporidium parvum gp60* subtypes reported in outbreaks. The zoonotic subtype IIaA15G2R1 was the most frequently reported, appearing in five different studies and multiple outbreaks.

*gp60* subtype	Outbreak setting/Vehicle/Source	Genotyping technique	Host	Country	Study period	Reference	Note
IIaA13G2R1	Multiple sources; zoonotic (vectors and direct contact) and anthroponotic routes of transmission	Sanger	Humans	Finland	2018	Suominen et al. (2023)	
IIaA14G1R1	Food-borne outbreak from self-pressed apple juice	Sanger	Humans	Norway	2018	Robertson et al. (2019)	
IIaA14G1R1r1	Zoonotic outbreak in assisted living home in 2022	Sanger	Humans	Sweden	2018–2022	Bujila et al. (2024)	
IIaA14R1	Unspecified cause outbreak at an agricultural school in 2022	Sanger	Humans	Sweden	2018–2022	Bujila et al. (2024)	
IIaA15G1R1	Food-borne outbreak from milk from on-farm dairy due to pasteurisation problems. Subtype found in a calf. Zoonotic outbreak (animal contact) from an open farm in 2015	Sanger	Cattle, Humans	England	2009–2017	Chalmers et al. (2019)	
IIaA15G1R1	Multiple sources; zoonotic (vectors and direct contact) and anthroponotic routes of transmission	Sanger	Humans	Finland	2018	Suominen et al. (2023)	
IIaA15G1R2	Zoonotic outbreaks (*n* = 3) (animal contact) from an open farm in 2013, 2015 (goat kid and lambs suspected), and 2016 (calf suspected, subtype not found in the calf, only IIaA17G1R1 found in the calf)	Sanger	Goat, Humans, Sheep	England	2009–2017	Chalmers et al. (2019)	
IIaA15G2R1	Water-borne outbreaks (*n* = 2) from a private drinking water supply in 2014, and a swimming pool in 2015. Zoonotic outbreaks (*n* = 13) (animal contact) from open farms in 2009, 2012 (*n* = 2) (lambs and goats suspected), 2013 (*n* = 2) (lambs and donkey suspected), 2014 (*n* = 2), 2015 (*n* = 3) (goat kid and lambs suspected in one of them), 2016 and 2017 (lambs suspected), agricultural college farm in 2016 (sheep and lambs suspected). Unknown vehicle outbreaks (*n* = 2) in an open prison in 2009 and in a community in 2015. Food-borne outbreak in 2012 (national, from ready-to-eat salad)	Sanger	Donkey, Goat, Humans, Sheep	England and Wales	2009–2017	Chalmers et al. (2019)	
IIaA15G2R1	Zoonotic outbreaks amongst veterinary students from direct contact with euthanised calves as part of fetotomy exercises. Three outbreaks in total; 2 from study period (September 2018-June 2019) and one suspected prior to study period (March 2018)	Sanger	Cattle: calves (euthanized), Humans (veterinary students)	Denmark	2018–2019	Thomas-Lopez et al. (2020)	
IIaA15G2R1	Water-borne outbreak affecting a family in 2014. Another outbreak in 2014 affecting a couple travelling together	Sanger	Humans	Sweden	2013–2014	Lebbad et al. (2021)	Mixed infection with IIaA14G2R1 in one of the cases and IIdA19G1 in another two cases. Most infections acquired during travel abroad
IIaA15G2R1	Food-borne national outbreak in 2022 (contaminated frisée salad) and zoonotic outbreak after school visit to farm in 2022 (calves)	Sanger	Humans	Sweden	2018–2022	Bujila et al. (2024)	
IIaA15G2R1	Multiple sources; zoonotic (vectors and direct contact) and anthroponotic routes of transmission	Sanger	Humans	Finland	2018	Suominen et al. (2023)	
IIaA15R1	Water-borne outbreak from a swimming pool (2014)	Sanger	Humans	England	2009–2017	Chalmers et al. (2019)	
IIaA16G1R1b	Outbreak amongst veterinary students in 2013	Sanger	Humans	Sweden	2013–2014	Lebbad et al. (2021)	Some of the patients became infected while travelling abroad
IIaA16G1R1b	Food-borne outbreaks (*n* = 2) at an event in 2019 (contaminated salad) and single cases in outbreaks involving an elderly home and two schools in 2021	Sanger	Humans	Sweden	2018–2022	Bujila et al. (2024)	
IIaA16G1R1b_variant	Zoonotic outbreak (animal contact) from an open farm	Sanger	Cattle: calves, Humans	Sweden	2015	Alsmark et al. (2018)	Sequence does not conform to naming convention by Robinson et al. (2025)
IIaA16G2R1	Food-borne outbreak from salad during a private dinner in 2013	Sanger	Humans	Sweden	2013–2014	Lebbad et al. (2021)	
IIaA16G3R1	Unspecified source single case infection in a female visitor from Germany (2020)	Sanger, NGS	Human	Australia	2019–2020	Braima et al. (2021)	Mixed infection with IIaA18G3R1
IIaA17G1R1	Zoonotic outbreaks (*n* = 12) (animal contact) from open farm in 2012 (*n* = 2), 2013 (*n* = 2), 2015 (*n* = 2) (lambs suspected and were also infected with the subtype IIaA21G3R1 in one of them), 2016 (calf suspected, one of the two subtypes in outbreak that was found in the calf) and 2017 (*n* = 3) (lambs suspected), open day on commercial farm in 2009 (calves and goats suspected) and at a college farm event in 2017. Unknown cause outbreak in 2014 in a school	Sanger	Cattle: calves, Goats: adult goats, kid; Humans, Sheep: lambs	England and Wales	2009–2017	Chalmers et al. (2019)	
IIaA17G1R1	Anthroponotic outbreak in 9 haemato-oncological in-patients in February 2020	Sanger	Humans: immunosuppressed patients	Slovakia	2019–2020	Hatalova et al. (2022)	
IIaA17G1R1c	Food-borne outbreak in a restaurant in 2020 (contaminated arugula)	Sanger	Humans	Sweden	2018–2022	Bujila et al. (2024)	
IIaA17G4R1	Epizootic diarrhoea outbreak across 181 farms between January 2021 and May 2022	Sanger	Cattle: pre-weaned calves	Republic of Korea	2021–2022	Jang et al. (2023)	
IIaA17R1	Food-borne outbreak during a private dinner in 2014	Sanger	Human	Sweden	2013–2014	Lebbad et al. (2021)	
IIaA18G1R1	Zoonotic outbreak (animal contact) from an open farm in 2016	Sanger	Humans	England	2009–2017	Chalmers et al. (2019)	
IIaA18G1R1	Multiple sources; zoonotic (vectors and direct contact) and anthroponotic routes of transmission	Sanger	Humans	Finland	2018	Suominen et al. (2023)	
IIaA18G1R1b_variant	Zoonotic outbreak (animal contact) from an open farm in 2015	Sanger	Cattle: calves, Humans	Sweden	2015	Alsmark et al. (2018)	Sequence does not conform to naming convention by Robinson et al. (2025)
IIaA18G1R1b_variant	Food-borne outbreak involving an elderly home and two schools in 2021 (contaminated kale)	Sanger	Humans	Sweden	2018–2022	Bujila et al. (2024)	
IIaA18G2R1	Zoonotic outbreaks (*n* = 2) (animal contact) from an open farm in 2013 (lamb was brought into school) and 2016 (suspected animal sources: lambs)	Sanger	Humans, Sheep: lambs	England and Wales	2009–2017	Chalmers et al. (2019)	
IIaA18G3R1	Unspecified source infection in female visitor (2020)	Sanger, NGS	Human	Australia	2019–2020	Braima et al. (2021)	Mixed infection with IIaA16G3R1. Subtype detected by NGS and not Sanger
IIaA18G3R1	Epizootic diarrhoea outbreak across 181 farms between January 2021 and May 2022	Sanger	Cattle: pre-weaned calves	Republic of Korea	2021–2022	Jang et al. (2023)	
IIaA18G3R1	Food-borne outbreaks from raw milk (2015, 2021)	TIDE	Humans	New Zealand	2010–2021	Garcia-R and Hayman (2023)	
IIaA18G3R1	Epizootic outbreak. Severe diarrhoea in six goat farms observed amongst goat kids during 2021–2023	Sanger	Goat: kids	Republic of Korea	2021–2023	Kim et al. (2023)	
IIaA19G1R1	Zoonotic outbreaks (*n* = 4) (animal contact) from an open farm in 2013 (suspected animal sources: lambs), 2014 and 2016 and in a commercial farm in 2013	Sanger	Humans, Sheep: lambs	England	2009–2017	Chalmers et al. (2019)	
IIaA19G4R1	Food-borne outbreaks from raw milk (2021)	TIDE	Humans	New Zealand	2010–2021	Garcia-R and Hayman (2023)	
IIaA20G3R1	Zoonotic outbreak (animal contact) from an open farm in 2015	Sanger	Humans	England	2009–2017	Chalmers et al. (2019)	
IIaA20G3R1	Epizootic diarrhoea outbreak across 181 farms between January 2021 and May 2022	Sanger	Cattle: pre-weaned calves	Republic of Korea	2021–2022	Jang et al. (2023)	
IIaA21G4R1	Outbreak from environmental water exposure during military exercises in 2014	Sanger	Humans	England	2009–2017	Chalmers et al. (2019)	
IIaA26G1R1^a^	Water-borne outbreak from a swimming pool in 2015	Sanger	Humans	England	2009–2017	Chalmers et al. (2019)	
IIdA15G1	Epizootic outbreaks in two goat farms, high morbidity and mortality (2018, 2019)	Sanger	Goat: kids	Republic of Korea	2019	Kim et al. (2022)	
IIdA15G1	Epizootic outbreak. Severe diarrhoea in six goat farms observed amongst goat kids during 2021–2023	Sanger	Goat: kids	Republic of Korea	2021–2023	Kim et al. (2023)	
IIdA16G1	Epizootic outbreak. Severe diarrhoea in six goat farms observed amongst goat kids during 2021–2023	Sanger	Goat: kids	Republic of Korea	2021–2023	Kim et al. (2023)	
IIdA17G1	Water-borne outbreak from a swimming pool in 2014	Sanger	Humans	England	2009–2017	Chalmers et al. (2019)	
IIdA19G1	Outbreak in 2014 affecting a couple travelling together	Sanger	Humans	Sweden	2013–2014	Lebbad et al. (2021)	Mixed infection with IIaA15G2R1 in two of the cases. Most infection cases occurred during travels abroad
IIdA19G1	Unspecified cause outbreak at agricultural school in 2022	Sanger	Humans	Sweden	2018–2022	Bujila et al. (2024)	
IIdA20G1e	Food-borne outbreak in 2019 (contaminated kale in Christmas buffet)	Sanger	Humans	Sweden	2018–2022	Bujila et al. (2024)	
IIdA21G1	Food-borne outbreaks (*n* = 3) at Christmas buffet (contaminated kale) in 2019, an elementary school (contaminated kale) in 2020 and single cases in outbreaks involving an elderly home and two schools in 2021 (contaminated kale).	Sanger	Humans	Sweden	2018–2022	Bujila et al. (2024)	
IIdA22G1	Zoonotic outbreak (animal contact) from an open farm in 2013	Sanger	Humans	England	2009–2017	Chalmers et al. (2019)	
IIdA22G1	Food-borne outbreak at a restaurant from parsley in 2014	Sanger	Humans	Sweden	2013–2014	Lebbad et al. (2021)	One patient acquired the infection from travelling abroad
IIdA22G1	Food-borne outbreak in 2019 (salad at private event)	Sanger	Humans	Sweden	2018–2022	Bujila et al. (2024)	
IIdA22G1c	Food-borne outbreaks (*n* = 2) nationally caused by the consumption of unpasteurised juice in 2019 and in an upper secondary school (contaminated buffet salad) in 2022	Sanger	Humans	Sweden	2018–2022	Bujila et al. (2024)	
IIdA23G1	Food-borne outbreak involving an elderly home and two schools in 2021 (contaminated kale)	Sanger	Humans	Sweden	2018–2022	Bujila et al. (2024)	
IIdA24G1	Zoonotic outbreak (animal contact) from an open farm in 2014. Food-borne national outbreak from sandwiches containing salad leaves from a coffee shop in 2015	Sanger	Humans	England	2009–2017	Chalmers et al. (2019)	
IIdA24G1	Outbreak amongst veterinary students in 2013	Sanger	Humans	Sweden	2013–2014	Lebbad et al. (2021)	Some of the patients became infected while travelling abroad
IIdA24G1	Water-borne swimming pool outbreak (2017)	TIDE	Humans	New Zealand	2010–2021	Garcia-R and Hayman (2023)	
IIdA24G1	National outbreak of unknown cause and source, 2019	Sanger	Humans	Sweden	2018–2022	Bujila et al. (2024)	

*Abbreviations*: *gp60*, 60 kDA glycoprotein; NGS, Next Generation Sequencing; TIDE, Tracking of Indels by Decomposition.

a Newly reported.

### 4.1 *Cryptosporidium hominis* and *C. parvum gp60* subtypes in humans

The genetic diversity and evolving epidemiology of *Cryptosporidium gp60* subtypes in humans have been a focus of extensive research due to their public health significance in outbreaks and sporadic cases (Chalmers et al., 2019; Bacchetti et al., 2023).

Globally, among humans, the most prevalent subtype families reported previously in *C. hominis* were Ia, Ib, Id, Ie and If, and in *C. parvum* IIa, IIc, and IId (Feng et al., 2018; Ryan et al., 2021b). This was also seen between the periods of December 2018 and January 2024 (Supplementary file 2), but examining beyond the subtype family level gives a more detailed picture of the diversity.

Historically, *C. hominis* subtype IbA10G2 and *C. parvum* subtype IIaA15G2R1 were the predominant causes of human cryptosporidiosis globally (Segura et al., 2015; Feng et al., 2018) with both linked to several outbreaks and sporadic cases in Europe, including the UK (Chalmers et al., 2019). Between 2009 and 2017, subtype IbA10G2 was linked to 32 anthroponotic, waterborne and foodborne outbreaks in humans, while IIaA15G2R1 was linked to 18 outbreaks including waterborne, zoonotic, and foodborne outbreaks in humans and animals (Chalmers et al., 2019).

However, the emergence and replacement of dominant subtypes in different regions is indicative of a dynamic epidemiological landscape shaped by environmental and anthropogenic factors, including the COVID-19 pandemic (Knox et al., 2021; Adamson et al., 2023; Bacchetti et al., 2023). In the USA, in the mid-2000’s, *C. hominis* IaA28R4 emerged following a multistate outbreak and quickly became widespread, replacing IbA10G2 as the dominant subtype (Xiao et al., 2009). However, by the early 2010’s, a further switch was taking place with IfA12G1R5, which began to dominate human infections in the USA after 2013 (Huang et al., 2023). In our review covering 2018–2024, we identified two reports of subtype IaA28R4, from China and Sweden, and five reports of IfA12G1R5, from Australia, New Zealand, Ireland, Canada and Sweden, compared to 25 reports of IbA10G2 in 16 different countries. Thus, while IfA12G1R5 has emerged, IbA10G2 remains widespread in terms of geography (Supplementary file 2). However, in recent years, IfA12G1R5 has replaced IbA10G2 in terms of prevalence in several countries, including the USA, Australia, New Zealand and the UK, which was not captured in our review as the publication came later (Braima et al., 2019; Huang et al., 2023; Peake et al., 2023; Adamson et al., 2023; Martinez et al., 2024). Similarly, although outside our review’s search window, a significant rise in cryptosporidiosis cases was observed in Spain in 2023 in all regions that were part of the surveillance network, with IfA12G1R5 detected in 62.3% of all case samples tested (Martinez et al., 2024). Other subtypes have also displaced IbA10G2, including IeA11G3T3, IdA16, IbA9G3 in Scotland and IbA12G3 in Australia (Braima et al., 2021; Bacchetti et al., 2023). These trends may be driven by environmental changes, human behavioural patterns, and ecological niche or host immune pressures, particularly following the COVID-19 pandemic (Bacchetti et al., 2023). For instance, the restrictions imposed during the COVID-19 pandemic affecting anthroponotic interactions (2020–2021), led to an absence of *C. hominis* notifications in New Zealand and distinct reduction the UK, while *C. parvum* transmission continued albeit with reduced numbers (Knox et al., 2021; Adamson et al., 2023). Interestingly, our review revealed a large number of published *gp60* subtypes across multiple WHO regions during this period (Fig. 2). Due to the pandemic, anthroponotic interactions and travel were significantly reduced, hygiene measures were enhanced, and some work processes slowed. These conditions may have provided researchers with more time to write up previous work and also compile region-specific surveillance reports.

For *C. parvum*, the IIaA15G2R1 subtype remains a dominant cause of zoonotic outbreaks globally, highlighting the critical role of livestock as reservoirs (Table 3). A study by Guy et al. (2021) demonstrated that, between 2008 and 2017, zoonotic transmission was the primary route of cryptosporidiosis in Canada, with *C. parvum* identified in 70.5% of the samples, and IIaA15G2R1 being the most prevalent subtype. Similarly, in England and Wales between 2009 and 2017, *C. parvum* accounted for all animal-related human outbreaks, predominantly caused by IIaA15G2R1 and IIaA17G1R1 (Chalmers et al., 2019). However, there are geographical differences. In Sweden, IIaA16G1R1 is commonly found in humans and livestock regardless of peaks caused by outbreaks (Alsmark et al., 2018; Lebbad et al., 2021; Bujila et al., 2024). Several outbreaks *via* food-borne, anthroponotic, and zoonotic routes occurred in Norway and Sweden in 2018 and 2022, caused by IIaA14G1R1 and IIaA14G1R1r1 (Robertson et al., 2019; Tipu et al., 2024; Bujila et al., 2024) (Table 3). In both Sweden and Scotland, IId family has been observed in human cryptosporidiosis in recent years, where the emergence of subtypes IIdA24G1 and the new subtypes IIdA7 and IIdA27G1_variant have been reported (Bacchetti et al., 2023; Bujila et al., 2024).

The IIc subtype family has been more commonly reported in low-to middle-income countries, along with other reportedly human-adapted families such as IIe and IIm (Feng et al., 2018; Ryan et al., 2021b). Various reports of IIc subtypes originate in Europe and Africa, with more subtype diversity revealed in various African countries (Supplementary file 2). A study in Lusaka, Zambia, identified IIcA5G3 as one of the most prevalent subtypes alongside the Ia and Ie families, with IIcA5G3a almost exclusively associated with human infections (Mulunda et al., 2020; Krumkamp et al., 2022). The latter has been identified as linked in evolutionary terms with humans (Kissinger, 2019; Nader et al., 2019)

Recent studies have also identified newly reported *C. parvum* subtype families (e.g. IIr, IIs, IIt, IIy, IIz, IIbeta, and IIgamma) in humans (Table 4), with several of these reported in intensive investigation of *gp60* subtypes in Sweden (Lebbad et al., 2021; Bujila et al., 2024; Robinson et al., 2025).

**Table 4:** Newly reported *C. hominis* and *C. parvum gp60* subtypes identified in humans between December 2018 and January 2024.

*gp60* subtype	Trinucleotide repeat	R repeat	Genotyping technique	GenBank ID	Country	Study period	Reference
IaA11R3	TCA	AAGACGGTGGTAAGG	Sanger	MT009623, OL598560	Colombia	NR	Uran-Velasquez et al. (2022)
IaA13R6	TCA	AAGACGGTGGTAAGG	Sanger	MN661180	Colombia	NR	Uran-Velasquez et al. (2022)
IaA16R3	TCA	AAGACGGTGGTAAGG	Sanger	MK331714	Thailand	1999–2004	Sannella et al. (2019)
IaA16R4	TCA	AAGACGGTGGTAAGG	Sanger	OL598578	Sweden	2018–2022	Bujila et al. (2024)
IdA11	TCA	NA	Sanger	MK331715	Thailand	1999–2004	Sannella et al. (2019)
ImA13G1	TCA/TCG	NA	Sanger	OP699729	Kenya	2022	Toriro et al. (2024)
IaA28R3	TCA	AAGACGGTGGTAAGG	Sanger	OL598538	Sweden	2018–2022	Bujila et al. (2024)
IIaA19G5R1	TCA/TCG	ACATCA	Sanger	MT009627, MT009628	Colombia		Uran-Velasquez et al. (2022)
IIaA19R1	TCA	ACATCA	Sanger	OL598555	Sweden	2018–2022	Bujila et al. (2024)
IIaA26G1R1	TCA/TCG	ACATCA	Sanger	MK391454	England	2009–2017	Chalmers et al. (2019)
IIeA11G1	TCA/TCG	NA	Sanger	MN904717, MN904721	Zambia	2017–2019	Mulunda et al. (2020)
IIeA13G1	TCA/TCG	NA	Sanger	KU852716	Sweden	2013–2014	Lebbad et al. (2021)
IIsA10G1	TCA/TCG	NA	Sanger	MN904704	Zambia	2017–2019	Mulunda et al. (2020)
IIaA12G1R1r1	TCA/TCG/ACA	ACATCA	Sanger	OR491776	Sweden	2018–2022	Bujila et al. (2024)
IIaA15G1R1_variant	TCA/TCG	ACATCA	Sanger	KU852704	Sweden	2013–2014	Lebbad et al. (2021)
IIaA15G1R1r1_variant	TCA/TCG/ACA	ACATCA	Sanger	OR491775	Sweden	2018–2022	Bujila et al. (2024)
IIaA17G2R1_variant	TCA/TCG	ACATCA	Sanger	OR491772	Sweden	2018–2022	Bujila et al. (2024)
IIaA20G1R1_variant	TCA/TCG	ACATCA/ACA	Sanger	OL598567	Sweden	2018–2022	Bujila et al. (2024)
IIdA7	TCA	NA	Sanger	OR491780	Sweden	2018–2022	Bujila et al. (2024)
IIdA27G1_variant	TCA/TCG	NA	Sanger	OL598571	Sweden	2018–2022	Bujila et al. (2024)
IIdA28G1	TCA/TCG	NA	Sanger	OL598551	Sweden	2018–2022	Bujila et al. (2024)
IIeA14G1	TCA/TCG	NA	Sanger	OR491779	Sweden	2018–2022	Bujila et al. (2024)
IInA10	TCA	NA	Sanger	KU852717	Sweden	2013–2014	Lebbad et al. (2021)
IIyA23G1R1^a^	TCA/TCG	ACATCA	Sanger	OL598564	Sweden	2018–2022	Bujila et al. (2024)
IIzA14R2^a^	TCA	ACATCA	Sanger	OL598569	Sweden	2018–2022	Bujila et al. (2024)
.A9R11^b^	TCA	ACATCA	Sanger	OP132396-OP132400	Egypt	2022	Ali et al. (2023)

*Abbreviations*: NA, not applicable; NR, not reported; *gp60*, 60 kDA glycoprotein.

a Does not conform to subtype naming nomenclature described in Robinson et al. (2025). It is not conventional to assign R repeats to the *C. parvum* IIy and IIz families.

b Subtype family could not be determined due to sequence length.

### 4.2 *Cryptosporidium hominis* and *C. parvum gp60* subtypes in livestock

Although our review identified fewer reports of *gp60* subtypes in livestock and non-human primates (NHPs) than humans (Table 1, Table 4, Table 5, Table 6; Supplementary file 2), these animals are important to understanding the impact of *Cryptosporidium* spp., as well as the zoonotic potential and transmission dynamics of these pathogens.

**Table 5:** Newly reported *C. hominis* and *C. parvum gp60* subtypes identified in livestock between December 2018 and January 2024.

*gp60* subtype	Trinucleotide repeat	R repeat	Genotyping technique	GenBank ID	Host	Country	Study period	Reference
IIaA11G3R1	TCA/TCG	ACATCA	Sanger	MN962678	Cattle	Türkiye	2016–2018	Kabir et al. (2020)
IIaA12G3R1	TCA/TCG	ACATCA	Sanger	MN962652, MN962659, MN962672, MN962675, MN962702-MN962703, MN962709, MN998537	Cattle, sheep	Türkiye	2016–2018	Kabir et al. (2020)
IIaA13G4R1	TCA/TCG	ACATCA	Sanger	MN962697	Sheep	Türkiye	2016–2018	Kabir et al. (2020)
IIaA24G1R1	TCA/TCG	ACATCA	Sanger	KX768790-KX768791	Cattle	Argentina	2013–2014	Lombardelli et al. (2019)
IIdA14G2R1^a^	TCA/TCG	ACATCG	Sanger	OP978554	Cattle	Belgium	2020–2021	Hoque et al. (2023)
IIdA21G2	TCA/TCG		Sanger	MT418848	Sheep	France	2017	Bordes et al. (2020)
IIlA21R2	TCA	ACATCA	Sanger	MH509214	Cattle	Estonia	2013–2014, 2015	Santoro et al. (2019)
IIdA24G2	TCA/TCG		Sanger	OR240215-OR240217	Cattle	China	2024	Qin et al. (2024)

*Abbreviation*: *gp60*, 60 kDA glycoprotein.

a Does not conform to subtype naming nomenclature described in Robinson et al. (2025). It is not conventional to assign R repeats to the *C. parvum* IId family.

Cryptosporidiosis in cattle mainly affects neonatal calves and is primarily caused by *C. parvum*, although infections with other species do occur, especially in older animals. Our review identified nearly 200 reports of *gp60* subtypes in cattle, which contrast with only 23 in sheep, even though contact with lambs was implicated in multiple human outbreaks (Table 3). As previously reported by others (Feng et al., 2018), the *C. parvum* families IIa and IId were the most commonly reported subtype families in livestock, particularly in neonatal calves, where they are associated with severe diarrhoea, significant morbidity and mortality. Reports of the IIa and IId subtype families show infections in a broad livestock range and across six and four WHO regions, respectively (Supplementary file 2). The IId family has a particularly strong association with dairy calves in China, where it has been reported to predominate (Li et al., 2019), which is supported by the results of our review where 70% of the reports of IId in livestock were from China, and only 11% from other Asian countries. We found no reports of IIa subtypes in livestock from China. Although not unique to China, subtype IIdA20G1 was particularly prominent there, linked to severe outbreaks in neonatal calves across various provinces with high mortality rates (Li et al., 2022; Zhang et al., 2022). Similarly, in China, there were several reports in livestock, and an outbreak in neonatal calves caused by IIdA19G1 (Li et al., 2019). Outside China, there was just a single report of this subtype in animals and five reports in humans. In contrast, numbers of reports of IId were lower in Europe and were largely absent in other WHO regions (Feng et al., 2017; Jia et al., 2022).

Amongst livestock, the IIaA15G2R1 subtype is one of the most frequently reported subtypes globally. This subtype has also been implicated in multiple human outbreaks, particularly those linked to animal-contact (Chalmers et al., 2019; Thomas-Lopez et al., 2020; Suominen et al., 2023) (Table 3), emphasizing the zoonotic risk posed by *C. parvum* from livestock sources. The IIaA17G2R1 subtype, the second most prevalent *C. parvum* subtype in a study by Guy et al. (2021) and one of the most frequently reported subtypes in this review, is recurrently found in calves and is strongly linked to farm visits and animal contact (Chalmers et al., 2011; Guy et al., 2021). It has also been reported in rodents in Thailand, suggesting that rats may serve as a secondary reservoir (Guy et al., 2021). A newly reported IIaA17G2R1_variant that has one of the TCG repeats in a different position within the microsatellite region compared to other IIaA17G2R1 subtypes, was identified by Bujila et al. (2024) in a sporadic human infection in Sweden. Another subtype, IIaA18G3R1, which has been identified in goat kids (Kim et al., 2023) and several reports in cattle (Supplementary file 2), has been prevalent in human cases in Ireland, where it was previously linked to waterborne outbreaks (De Waele et al., 2013). The overlap of genetic subpopulations between livestock and humans suggests that these animals can contribute to the transmission of specific subtypes, particularly in settings such as petting farms where human-animal interactions are frequent (Chalmers et al., 2019) or where transmission *via* water is common (De Waele et al., 2013) (Table 5).

### 4.3 *Cryptosporidium hominis* and *C. parvum gp60* subtypes in non-human primates (NHPs)

Recent studies into other hosts such as non-human primates (NHPs), have helped expand the understanding of their potential for zoonotic transmission of *Cryptosporidium* spp*.* NHPs, known to share both habitats and genetic similarities with humans, have become important targets of One Health surveillance schemes due to their role as reservoirs for a various range of pathogens (Balansard et al., 2019; Hailu et al., 2022). Their genetic similarity to humans places them at a critical vantage point in understanding zoonotic transmission and this makes the identification of anthroponotic *C. hominis* in NHPs concerning (Huang et al., 2024).

Eight *C. hominis* families (Ia, Ib, Id, Ie, Ii, Im, In, and Io) and three *C. parvum* families (IIa, IId, and IIo) were reported in NHPs in Ethiopia and China (Supplementary file 2). Ten newly reported *C. hominis gp60* subtypes were identified in NHPs, as others have been previously identified in other hosts including humans and livestock. The identification of previously identified zoonotic *C. parvum* subtypes in NHPs, suggests that these animals, as well as livestock, pose a potential zoonotic risk to humans (Shu et al., 2022). Other *C. parvum* subtype families, such as IIo and IIp, have previously been reported to be more host-adapted to NHPs (Zhang et al., 2019; Jia et al., 2022), although two subtypes in the IIo family were recently reported in humans (Sannella et al., 2019; Garcia-R et al., 2020). The current lack of reports of IIp in humans may be due to a lack of exposure rather than infectivity.

A study of long-tailed macaques and rhesus macaques in China found that *C. hominis* was the most prevalent species, demonstrating the potential for zoonotic transmission of this predominantly human-infecting species from these primates to humans and *vice versa* (Zhao et al., 2019). Chen et al. (2019) identified a newly reported *C. hominis* subtype family with genetic similarities to the family Ia in farmed crab-eating macaques in Guangxi, China. This subtype was initially misclassified as IdA14, but later reclassified as the family In.

Several of the *C. hominis* families discovered in NHPs, such as Ii, Ik, Im, and In, have been considered as animal-adapted (Widmer et al., 2020). However, an outbreak amongst British military personnel in Kenya was caused by the *C. hominis* subtype ImA13G1, a novel subtype of the Im family (Toriro et al., 2024). In 2014, two related people from Sweden who had been on a trip to a monkey farm in Thailand were infected with the *C. hominis* subtype IiA17 (Lebbad et al., 2018). These reports highlight the potential role of NHPs as reservoirs for human infections, particularly in areas with frequent human-NHP interactions or where environmental or water contamination may occur.

In 2024, Huang and colleagues reported the *C. hominis* IbA12G3 subtype in a monkey, noting that this variant was genetically distinct from the human isolate by approximately 15,324 core SNPs across the genome. Comparative genomics indicated the monkey *gp60* variant is genetically similar to the NHP-adapted ImA20 subtype and the newly reported InA17 subtype in most genomic regions, with sequence introgression from human IbA12G3 at a few loci, including the *gp60* locus. This discovery highlights the potential for genetic recombination between human-adapted and NHP-adapted subtypes, which may lead to the emergence of unique variants with implications for cross-species transmission (Huang et al., 2024).

Also using comparative genomics, Huang et al. (2024) demonstrated that while genetic similarities exist among host-adapted *C. hominis* variants, isolates from humans, equines, and macaques formed distinct clades. They possess highly divergent genomes with minimal gene flow between clades, highlighting differing host adaptation within *C. hominis*. These findings reinforce the value of whole-genome sequencing (WGS) over single-locus typing for understanding the genetic dynamics of *Cryptosporidium* subtypes (Table 6).

**Table 6:** Newly reported *C. hominis* and *C. parvum gp60* subtypes identified in NHPs between December 2018 and January 2024.

*gp60* subtype	Trinucleotide repeat	R repeat	Genotyping technique	GenBank ID	Host	Country	Study period	Reference
IaA20R3a	TCA/TCG	AAGACGGTGGTAAGG	Sanger	MK270518	Crab-eating macaque	China	2019	Zhao et al. (2019)
ImA18	TCA		Sanger	MG952710	Crab-eating macaque	China	2016–2018	Chen et al. (2019)
InA14	TCA		Sanger	MG952714	Crab-eating macaque	China	2016–2018	Chen et al. (2019)
InA17	TCA		Sanger	MG952713	Crab-eating macaque	China	2016–2018	Chen et al. (2019)
InA23	TCA		Sanger	OQ032496, OQ243227	Crab-eating macaque, Rhesus macaques	China	2022	Zhang et al. (2023)
InA24	TCA		Sanger	OQ032497, OQ243228	Crab-eating macaque, Rhesus macaques	China	2022	Zhang et al. (2023)
InA25	TCA		Sanger	OQ032494, OQ243225	Crab-eating macaque, Rhesus macaques	China	2022	Zhang et al. (2023)
InA26	TCA		Sanger	MG952711	Crab-eating macaque	China	2016–2018	Chen et al. (2019)
IoA17a	TCA		Sanger	MK270519	Crab-eating macaque	China	2019	Zhao et al. (2019)
IoA17b	TCA		Sanger	MK270520	Crab-eating macaque	China	2019	Zhao et al. (2019)

*Abbreviations*: NHP, non-human primates; *gp60*, 60 kDA glycoprotein.

### 4.4 Study limitations

Our literature review was limited by reliance on a single database and three keywords. Findings that occurred within this time frame that were not published by 31st January 2024 were not captured in this review nor were *gp60* reports that were not included in the title or keywords of the paper. Additionally, reports of *gp60* subtypes are affected by prospective sampling strategies or diagnostic testing, subtyping and reporting practices in different countries. The findings are therefore subject to reporting bias. The results of this study may have been impacted by the restrictions to control the COVID-19 pandemic, limiting the activities of some researchers who may have been more prolific in publishing results in 2020 and 2021, or diverting others to tasks restricting their *Cryptosporidium* reporting during the period.

## 5 Conclusions, future challenges and multilocus typing

The *gp60* gene has become a widely employed marker for the discrimination within *Cryptosporidium* species, providing insights into intra-species diversity and zoonotic transmission (Li et al., 2021). This review has identified and updated the record of that diversity for *C. hominis* and *C. parvum* in humans, livestock and NHPs. The accuracy of *gp60* subtype reporting can be variable, but newly published guidance will help improve this. However, the highly polymorphic nature of *gp60*, coupled with selective pressures arising from the parasites’ sexual recombination, poses challenges to its consistent use as a single subtype marker (Feng et al., 2018). Despite providing some epidemiological utility, the full genetic diversity of *Cryptosporidium* species has yet to be resolved. A globally standardised multilocus scheme would enable more accurate characterisation, phylogenetic and epidemiological analysis of these clinically important parasites. Future characterisation and molecular epidemiology will almost certainly rely on genomic analysis, but while the development of tools and techniques for *Cryptosporidium* spp. are progressing, routine application is not yet widely available and *gp60* typing will remain a useful tool to compare the global diversity of *Cryptosporidium*.

## CRediT authorship contribution statement

**Deborah B. Oladele:** Conceptualization, Investigation, Data curation, Visualization, Writing – original draft, Writing – review & editing. **Martin Swain:** Methodology, Supervision, Funding acquisition, Writing – review & editing. **Guy Robinson:** Writing – review & editing, Funding acquisition. **Amanda Clare:** Writing – review & editing, Supervision. **Rachel M. Chalmers:** Conceptualization, Funding acquisition, Writing – review & editing.

## Ethical approval

Not applicable.

## Funding

This work was supported by the Natural Environment, Biotechnology and Biological Sciences and Medical Research councils (10.13039/100008668NERC, 10.13039/501100000268BBSRC and 10.13039/501100000265MRC) (grant number: NE/X016714/1) as part of the One Health for One Environment: An A-Z Approach for Tackling Zoonoses (‘OneZoo’) Centre for Doctoral Training.

## Declaration of competing interests

The authors declare that they have no known competing financial interests or personal relationships that could have appeared to influence the work reported in this paper.

## Data Availability

The data supporting the conclusions of this article are included within the article and its supplementary files.

## Appendix A Supplementary data

Multimedia component 1 Supplementary file 1. Screening criteria and selected studies. Multimedia component 1

Multimedia component 2 Supplementary file 2**.** Master list of all reported *C. hominis* and *C. parvum gp60* subtypes between December 2018 and January 2024 along with data categorization. **Worksheet 1.** Master list. Subtypes with ∗ were found in different outbreaks in the same study. **Worksheet 2.** List of *gp60* subtypes reported. **Worksheet 3.***gp60* subtypes reported by WHO region and country. **Worksheet 4.** Most frequently reported subtypes by WHO region. **Worksheet 5.***gp60* subtypes families reported. **Worksheet 6**: Newly-reported *gp60* subtypes. **Worksheet 7.***gp60* subtypes reported in outbreaks. **Worksheet 8:***gp60* subtypes reported by publication year (December 2018 to January 2024). Multimedia component 2
