# Supplementary material for: A review of recent Cryptosporidium hominis and Cryptosporidium parvum gp60 subtypes
Source: Curr Res Parasitol Vector Borne Dis. 2025 Jul 6;8:100292. doi: 10.1016/j.crpvbd.2025.100292 (PMC12304688; doi:10.1016/j.crpvbd.2025.100292)
Supplement: Multimedia component 1 — Supplementary file 1. Screening criteria and selected studies. [file mmc1.pdf]

Supplementary file 1

Screening of 222 papers from PUBMED from December 2018 to January 2024.

Search terms: (CRYPTOSPORIDIUM) AND ((GP60) OR (60 KDA GLYCOPROTEIN))

Level 1:

Included: 149 papers focusing on epidemiological data and molecular characterization of *Cryptosporidium gp60* subtypes in humans, livestock and non-human primates were included. Livestock were defined according to Britannica's definition (Britannica, 2019), with additional inclusion of alpacas, rabbits, and poultry. The livestock considered were cattle, sheep, pigs, goats, horses, donkeys, mules, deer, buffalo, oxen, llamas, camels, poultry, alpacas, and rabbits. Studies involving reptiles, fish, rodents, squirrels, pets (cats, dogs, hamsters, guinea pigs, ponies), pigeons, crows, minks, and ostriches were excluded.

Excluded: 73 papers excluded.

- Studies with hosts aside the ones mentioned in inclusion criteria.
- Publications focused solely on the development of newly-reported protocols, diagnostics, or molecular assays without primary or secondary occurrence or epidemiological data were excluded.
- Studies concentrating exclusively on experimental parasitology or molecular biology without *gp60* subtype identification *Cryptosporidium* were also excluded.
- Studies that solely examined environmental samples were excluded.

Level 2:

Included: 128 papers focusing or reporting cases with *C. parvum* or *C. hominis* identified.
Excluded: 21 papers.

Level 3:

Included: 123 papers with primary analysis.

- **Excluded:** 5 meta-analysis/systematic reviews were removed (37941927, 36738819, 35065302, 34337169, 30621759).
-

Additional exclusion: 4 papers met the criteria above but were excluded:

- Papers that could not be accessed due to full article being in another language and closed access (31713374)
- Studies not containing any gp60 subtypes either due to absence of *C. parvum* or *C. hominis gp60* subtypes in our selected hosts or were not able to successfully amplify/genotype any of the sequence (35059456. 33142151, 37152690)

Pubmed IDs are organized in descending order by publication year in the same order it appears in the master list (Supplementary file 2, table 1).

	PMID (222)	Level 1	Level 2	Level 3	Additional Exclusion
1	38915888	Yes	Yes	Yes	Yes
2	38660488	No	No	No	No
3	38242277	Yes	Yes	Yes	Yes
4	38188480	No	No	No	No
5	38291399	Yes	Yes	Yes	Yes
6	38318169	No	No	No	No
7	38275800	No	No	No	No
8	38250201	Yes	Yes	Yes	Yes
9	38165486	Yes	Yes	Yes	Yes
10	39637311	No	No	No	No
11	39353100	Yes	Yes	Yes	Yes
12	38949636	No	No	No	No
13	38654951	Yes	Yes	Yes	Yes
14	38199683	Yes	Yes	Yes	Yes
15	38116366	Yes	Yes	Yes	Yes
16	38052995	Yes	Yes	Yes	Yes
17	37792051	Yes	Yes	Yes	Yes
18	37604363	No	No	No	No
19	38029266	No	No	No	No
20	37941927	Yes	Yes	No	No
21	37462744	Yes	Yes	Yes	Yes
22	37347287	Yes	Yes	Yes	Yes
23	37347286	Yes	No	No	No
24	37150475	No	No	No	No
25	37701073	Yes	Yes	Yes	Yes
26	37626358	Yes	Yes	Yes	Yes
27	37454101	No	No	No	No
28	37483300	No	No	No	No
29	37886251	No	No	No	No

30	37326984	Yes	Yes	Yes	Yes
31	37349848	Yes	Yes	Yes	Yes
32	37030025	Yes	Yes	Yes	Yes
33	36938817	Yes	Yes	Yes	Yes
34	37252910	Yes	Yes	Yes	Yes
35	36959486	No	No	No	No
36	36533513	No	No	No	No
37	35680650	No	No	No	No
38	37152690	Yes	Yes	Yes	No
39	36738819	Yes	Yes	No	No
40	37026096	Yes	Yes	Yes	Yes
41	36739387	Yes	Yes	Yes	Yes
42	36974018	No	No	No	No
43	36539638	Yes	Yes	Yes	Yes
44	36526925	Yes	No	No	No
45	36266958	Yes	Yes	Yes	Yes
46	36205770	Yes	No	No	No
47	36436893	Yes	Yes	Yes	Yes
48	35908575	No	No	No	No
49	36231975	No	No	No	No
50	36694568	Yes	Yes	Yes	Yes
51	36018393	No	No	No	No
52	35906999	Yes	No	No	No
53	36095017	Yes	Yes	Yes	Yes
54	35857091	No	No	No	No
55	35840104	Yes	Yes	Yes	Yes
56	35545736	No	No	No	No
57	36031635	Yes	Yes	Yes	Yes
58	36072226	No	No	No	No
59	36015058	No	No	No	No
60	35865179	Yes	No	No	No
61	35842437	No	No	No	No
62	35883352	Yes	Yes	Yes	Yes
63	35802653	Yes	Yes	Yes	Yes
64	35873143	Yes	Yes	Yes	Yes
65	35794543	Yes	Yes	Yes	Yes
66	36466031	Yes	Yes	Yes	Yes
67	35488924	Yes	Yes	Yes	Yes
68	35668467	Yes	No	No	No
69	35498551	No	No	No	No
70	35454277	Yes	Yes	Yes	Yes
71	35421188	Yes	Yes	Yes	Yes
72	35302613	No	No	No	No

73	35416875	Yes	Yes	Yes	Yes
74	35255909	No	No	No	No
75	34388242	Yes	Yes	Yes	Yes
76	39170593	Yes	Yes	Yes	Yes
77	35065302	Yes	Yes	No	No
78	35336110	Yes	Yes	Yes	Yes
79	35202344	No	No	No	No
80	34417806	Yes	Yes	Yes	Yes
81	34499298	No	No	No	No
82	35100286	Yes	Yes	Yes	Yes
83	35059456	Yes	Yes	Yes	No
84	36350192	Yes	No	No	No
85	35012721	Yes	Yes	Yes	Yes
86	34735628	Yes	Yes	Yes	Yes
87	34730381	No	No	No	No
88	34113708	No	No	No	No
89	34835519	Yes	Yes	Yes	Yes
90	34780521	Yes	Yes	Yes	Yes
91	34663327	No	No	No	No
92	34417865	Yes	No	No	No
93	34182122	No	No	No	No
94	34683387	Yes	No	No	No
95	34683369	No	No	No	No
96	34547101	No	No	No	No
97	34398925	No	No	No	No
98	34343188	No	No	No	No
99	34337169	Yes	Yes	No	No
100	33971308	Yes	No	No	No
101	33848684	Yes	Yes	Yes	Yes
102	33819572	Yes	Yes	Yes	Yes
103	34474801	Yes	Yes	Yes	Yes
104	34202513	Yes	No	No	No
105	34203099	Yes	No	No	No
106	34115820	Yes	Yes	Yes	Yes
107	34044353	No	No	No	No
108	33992605	No	No	No	No
109	33748443	No	No	No	No
110	33455078	Yes	No	No	No
111	34070669	No	No	No	No
112	33926039	Yes	Yes	Yes	Yes
113	32150243	Yes	Yes	Yes	Yes
114	35284902	No	No	No	No
115	33879230	No	No	No	No

116	33921541	No	No	No	No
117	33918893	Yes	Yes	Yes	Yes
118	33421439	No	No	No	No
119	33805766	Yes	Yes	Yes	Yes
120	33259769	Yes	Yes	Yes	Yes
121	33298606	Yes	No	No	No
122	33543733	No	No	No	No
123	33246242	No	No	No	No
124	33137502	Yes	Yes	Yes	Yes
125	33482898	Yes	Yes	Yes	Yes
126	33498291	No	No	No	No
127	33355530	Yes	Yes	Yes	Yes
128	33276616	No	No	No	No
129	33142151	Yes	Yes	Yes	No
130	33084542	Yes	No	No	No
131	33057813	Yes	Yes	Yes	Yes
132	33010364	Yes	Yes	Yes	Yes
133	32589940	Yes	Yes	Yes	Yes
134	33318958	Yes	Yes	Yes	Yes
135	33324584	Yes	No	No	No
136	33202835	Yes	Yes	Yes	Yes
137	33009948	No	No	No	No
138	32996049	Yes	Yes	Yes	Yes
139	32858044	Yes	Yes	Yes	Yes
140	32772945	No	No	No	No
141	32504804	Yes	Yes	Yes	Yes
142	32709724	Yes	Yes	Yes	Yes
143	32955234	Yes	Yes	Yes	Yes
144	32899825	Yes	Yes	Yes	Yes
145	32594239	Yes	Yes	Yes	Yes
146	32473987	Yes	Yes	Yes	Yes
147	32302466	No	No	No	No
148	32842484	Yes	Yes	Yes	Yes
149	32974408	Yes	Yes	Yes	Yes
150	32811542	Yes	Yes	Yes	Yes
151	32903723	Yes	No	No	No
152	32640465	Yes	Yes	Yes	Yes
153	32612927	Yes	Yes	Yes	Yes
154	32722048	Yes	No	No	No
155	32641157	No	No	No	No
156	32862916	No	No	No	No
157	32494897	Yes	Yes	Yes	Yes
158	32368610	Yes	Yes	Yes	Yes

159	31978845	Yes	Yes	Yes	Yes
160	32204732	No	No	No	No
161	32004534	No	No	No	No
162	32053684	Yes	Yes	Yes	Yes
163	31973771	No	No	No	No
164	31910816	Yes	No	No	No
165	33206594	Yes	Yes	Yes	Yes
166	33141660	Yes	Yes	Yes	Yes
167	33048665	Yes	Yes	Yes	Yes
168	32584252	Yes	Yes	Yes	Yes
169	32428181	Yes	No	No	No
170	31758296	No	No	No	No
171	31452160	No	No	No	No
172	31452159	No	No	No	No
173	31796198	Yes	Yes	Yes	Yes
174	31796189	Yes	Yes	Yes	Yes
175	31700790	No	No	No	No
176	31667080	Yes	Yes	Yes	Yes
177	31624910	Yes	Yes	Yes	Yes
178	31295579	Yes	Yes	Yes	Yes
179	31671699	Yes	Yes	Yes	Yes
180	31666095	Yes	No	No	No
181	31661498	Yes	Yes	Yes	Yes
182	31661007	No	No	No	No
183	31713374	Yes	Yes	Yes	No
184	31715696	Yes	Yes	Yes	Yes
185	31437689	Yes	Yes	Yes	Yes
186	31540508	No	No	No	No
187	31442890	Yes	Yes	Yes	Yes
188	31327324	Yes	Yes	Yes	Yes
189	31194962	Yes	Yes	Yes	Yes
190	31146044	Yes	Yes	Yes	Yes
191	31341769	No	No	No	No
192	31338292	No	No	No	No
193	30974206	Yes	Yes	Yes	Yes
194	31307508	Yes	Yes	Yes	Yes
195	31303210	Yes	Yes	Yes	Yes
196	31303202	No	No	No	No
197	31187226	Yes	Yes	Yes	Yes
198	30826667	No	No	No	No
199	31156581	No	No	No	No
200	30833731	Yes	Yes	Yes	Yes
201	30790038	Yes	Yes	Yes	Yes

202	31039801	Yes	Yes	Yes	Yes
203	31027593	Yes	Yes	Yes	Yes
204	30738722	Yes	Yes	Yes	Yes
205	30590124	No	No	No	No
206	30192924	Yes	Yes	Yes	Yes
207	30867023	Yes	Yes	Yes	Yes
208	30867022	Yes	Yes	Yes	Yes
209	30808303	Yes	Yes	Yes	Yes
210	30807710	No	No	No	No
211	30240872	No	No	No	No
212	30086806	Yes	Yes	Yes	Yes
213	30621759	Yes	Yes	No	No
214	31599539	Yes	Yes	Yes	Yes
215	30869057	Yes	Yes	Yes	Yes
216	30179310	Yes	Yes	Yes	Yes
217	30503096	No	No	No	No
218	30501684	Yes	Yes	Yes	Yes
219	31014741	Yes	Yes	Yes	Yes
220	31014737	No	No	No	No
221	30207199	Yes	Yes	Yes	Yes
222	30055334	Yes	Yes	Yes	Yes
